# PSMA PET total tumor volume predicts outcome of patients with advanced prostate cancer receiving [^177^Lu]Lu-PSMA-617 radioligand therapy in a bicentric analysis

**DOI:** 10.1007/s00259-020-05040-1

**Published:** 2020-09-24

**Authors:** Robert Seifert, Katharina Kessel, Katrin Schlack, Manuel Weber, Ken Herrmann, Maximilian Spanke, Wolfgang P. Fendler, Boris Hadaschik, Jens Kleesiek, Michael Schäfers, Matthias Weckesser, Martin Boegemann, Kambiz Rahbar

**Affiliations:** 1 Department of Nuclear Medicine, University Hospital Münster, Albert-Schweitzer-Campus 1, D-48149 Münster, Germany; 2 Department of Nuclear Medicine, University Hospital Essen, Essen, Germany; 3 West German Cancer Center (WTZ), Münster and Essen, Germany; 4 German Cancer Consortium (DKTK), Essen, Germany; 5 Department of Urology, University Hospital Münster, Münster, Germany; 6 Department of Urology, University Hospital Essen, Essen, Germany; 7 Division of Radiology, German Cancer Research Center, Heidelberg, Germany

**Keywords:** PSMA, PET-CT, Total tumor volume, Lu-PSMA, mCRPC

## Abstract

**Introduction:**

[^177^Lu]Lu-PSMA-617 (Lu-PSMA) radioligand therapy is an emerging treatment option for patients with end-stage prostate cancer. However, response to Lu-PSMA therapy is only achieved in approximately half of patients. It is clinically important to identify patients at risk of poor outcome. Therefore, the aim of this study was to evaluate pretherapeutic PSMA PET derived total tumor volume and related metrics as prognosticators of overall survival in patients receiving Lu-PSMA therapy.

**Methods:**

A total number of 110 patients form the Departments of Nuclear Medicine Münster and Essen were included in this retrospective analysis. Baseline PSMA PET-CT was available for all patients. Employing a previously published approach, all tumor lesions were semi-automatically delineated in PSMA PET-CT acquisitions. Total lesion number, total tumor volume (PSMA-TV), total lesion uptake (PSMA-TLU = PSMA-TV * *SUV*_mean_), and total lesion quotient (PSMA-TLQ = PSMA-TV / *SUV*_mean)_ were quantified for each patient. Log2 transformation was used for regressions.

**Results:**

Lesion number, PSMA-TV, and PSMA-TLQ were prognosticators of overall survival (HR = 1.255, *p* = 0.009; HR = 1.299, *p* = 0.005; HR = 1.326, *p* = 0.002). In a stepwise backward Cox regression including lesion number, PSMA-TV, PSA, LDH, and PSMA-TLQ, only the latter two remained independent and statistically significant negative prognosticators of overall survival (HR = 1.632, *p* = 0.011; HR = 1.239, *p* = 0.024). PSMA-TLQ and LDH were significant negative prognosticators in multivariate Cox regression in contrast to PSA value.

**Conclusion:**

PSMA-TV was a statistically significant negative prognosticator of overall survival in patients receiving Lu-PSMA therapy. PSMA-TLQ was an independent and superior prognosticator of overall survival compared with PSMA-TV.

## Introduction

The management of end-stage metastatic prostate cancer is challenging, as only limited therapeutic options are present [1]. However, [^177^Lu] Lutetium PSMA-617 (Lu-PSMA) radioligand therapy has become a promising treatment option in end-stage prostate cancer and is currently employed after failure of androgen deprivation therapy, next generation androgen receptor targeted therapy (ARTA), and taxane chemotherapy [2, 3]. It offers a favorable tolerability, and the efficacy is currently evaluated in prospective trials, including a head-to-head comparison against cabazitaxel [4–7].

Biochemical response (PSA decline by at least 50% from baseline) of patients undergoing Lu-PSMA therapy is achieved in 45–66% of cases [2, 3, 8]. Therefore, the identification of biomarkers that are associated with response to Lu-PSMA therapy and outcome is of great importance, as this would enable early management changes.

It is well known that a high volume of disease is a negative prognostic factor of patients with prostate cancer [9, 10]. For example, the volumetric bone scan index, which is measuring the volumetric affection of the skeleton by metastases, was an excellent prognosticator of overall survival in prostate cancer patients [11]. As Lu-PSMA therapy is applied in fixed doses and not in a disease extent adapted fashion, the tumor volume could be even more predictive of overall survival. However, there are contradictory reports on the prognostic value of the total tumor volume for patients treated by Lu-PSMA therapy: Ferdinandus et al. had not observed a statistically significant correlation between PSMA-TV and overall survival in 50 patients treated with Lu-PSMA [12]. Yet, a preliminary analysis by Seifert et al. found PSMA-TV to be significantly correlated with the overall survival in patients treated with Lu-PSMA [13].

It might be of clinical importance to evaluate the prognostic value of PSMA-TV for Lu-PSMA patients, as PSMA-TV could potentially be employed to anticipate the need of therapy intensification. Therefore, the aim of this study was to evaluate if PSMA PET derived tumor volume parameters can prognosticate the overall survival of patients treated with Lu-PSMA therapy. To this end, patients from two departments, which had partly been included in previous analyses, were included to specifically evaluate the relevance of the total tumor volume and related parameters.

## Methods

### Patients and eligibility for [^177^Lu]Lu-PSMA-617 therapy

All patients referred to Lu-PSMA therapy in either Essen or Münster between December 2014 and November 2018 who underwent baseline PSMA-11 PET-CT prior to Lu-PSMA therapy were included in this retrospective analysis. Patients were eligible for therapy, if they met the following inclusion criteria: progressive, metastatic castration-resistant prostate cancer (mCRPC), history of chemotherapy (docetaxel or cabazitaxel) if no contraindications were present, PSMA avid tumor lesions, adequate hematological reserve and adequate liver, as well as renal function parameters [14].

Decision for Lu-PSMA therapy was done on a case-by-case basis in the interdisciplinary tumor boards due to clinical indication. Data analysis was done retrospectively. The retrospective analysis of the Münster cohort was approved by the local ethics committee (No. 2016-585-f-S, Ethikkommission der Ärztekammer Westfalen-Lippe und der Westfälischen Wilhelms-Universität Münster). The retrospective analysis of the Essen cohort was approved by the local ethics committee (No. 19-8570-BO). The Münster cohort was previously employed for an analysis of SUV metrics [15]. The previously reported patient cohort (*n* = 40) that was used for a preliminary technical analysis is also included in this study [13]. Patients from Essen have been reported previously in a study investigating diffuse bone marrow involvement [16].

### Administration of [^177^Lu] Lutetium PSMA-617 therapy

The PSMA-617 precursor was provided by ABX (ABX GmbH, Radeberg, Germany). [^177^Lu] Lutetium was provided by ITG Isotopes Technology, Garching, Germany. Syntheses of [^177^Lu]Lu-PSMA-617 (Lu-PSMA) was done as described elsewhere [17]. Lu-PSMA was administered every 8 weeks (median 8.0 weeks [2.6]) in a medium dose of 6.7 GBq [1.2] until disease progression, severe adverse reactions, altered therapy regime, or death.

### PSMA PET imaging

The PSMA-11 precursor was obtained from ABX (ABX GmbH, Radeberg, Germany). Image acquisition was initiated 60 min after tracer administration. A Biograph mCT was used for image acquisition (Siemens Healthineers, Knoxville, TN, USA). Median time from PET acquisition until Lu-PSMA therapy start was 32 days [18]. PET reconstruction was done using manufacture standard tools (with iterative reconstruction and time of flight correction, but without point spread function adjustments).

### PET image analysis

A research software prototype was used for image analysis (MIWBAS, version 1.0, Siemens Medical Solutions USA, Inc., Knoxville, TN, USA). Semi-automated PSMA PET lesion delineation was done as described elsewhere [13]. For segmentation, a liver specific threshold was employed: threshold = (4.3 / liver *SUV*_mean_) * (liver *SUV*_mean_ + liver *SUV*_standard deviation_). Briefly, all metastases with an *SUV*_peak_ greater than the liver specific threshold were segmented. Delineable lesions with PSMA uptake lesser than the liver specific threshold were manually added, if necessary (in 7 patients). Lesions smaller than 0.5 ml were discarded.

Segmentation of each individual lesion was done by calculating a lesion specific threshold, which was defined as 50% of the maximum local SUV. The volume of a segmented lesion was denoted TV. The volumes of all lesions are summed to obtain the whole-body PSMA-TV for each patient (Eq. 1) [13]:

1 $$ PSMA- TV=\sum \limits_{\mathrm{lesions}} TV $$

In analogy to FDG total lesion glycolysis, the TV of each lesion was multiplied by its *SUV*_mean_. The resulting products are summed to obtain the whole-body PSMA-TLU for each patient (Eq. 2) [19]:

2 $$ PSMA- TLU=\sum \limits_{\mathrm{lesions}} TV\ast {SUV}_{\mathrm{mean}} $$

PSMA-TV is suspected to be a negative prognosticator of survival, whereas *SUV*_mean_ is suspected to be a positive prognosticator; both effects may antagonize in the PSMA-TLU biomarker [12, 13]. Therefore, the TV of each lesion was divided by its *SUV*_mean_. The resulting quotients are summed to obtain the whole-body PSMA-TLQ for each patient (Eq. 3):

3 $$ PSMA- TLQ=\sum \limits_{\mathrm{lesions}}\frac{TV}{SUV_{\mathrm{mean}}} $$

### Statistical analysis

SPSS version 25 was used for descriptive statistics, univariate and multivariate (Cox-) regression, spearman correlation, and Mann Whitney *U* tests (IBM Corp., Armonk, NY, USA). For stepwise Cox regression, a backward LR approach with standard settings of SPSS was employed. Logarithmic transformation (base 2, log transformed) was used for regression analyses. R version 3.5.2 was used to find ideal cutoffs for survival stratification, log rank tests, and Kaplan Meier curves [20, 21]. The log-log option was used to calculate the confidence intervals for overall survival time. Values are presented as median with inter quartile range in squared brackets. *H*_0_ was rejected if *p* < 0.05.

## Results

### Patient characteristics

All patients were castration resistant and received androgen deprivation therapy; 97.3% had received prior next generation antiandrogen treatment (enzalutamide and/or abiraterone). Eighty-one percent of patients had a history of docetaxel treatment (26% of cabazitaxel treatment). Detailed patient characteristics are given by Table 1.

**Table 1:** Patient characteristics

Patient characteristics	Total cohort
Number of patients	110
Patients from Essen/Münster	25/85
Age	72 [11.1]
Gleason score	8 (range: 5–10)
Metastases locations	
Bone	102 (92.7%)
Lymph nodes	84 (76%)
Liver	26 (24%)
Lung/pleura	24 (22%)
History of previous therapies	
Abiraterone	92 [83.6%]
Enzalutamide	90 [81.8%]
Docetaxel	92 [80.9%]
Cabazitaxel	29 [26.4%]
Pre- Lu-PSMA therapy blood parameter	
Prostate-specific antigen [ng/ml]	231.0 [587.5]
Lactate dehydrogenase [U/l]	317.0 [245.0]
Aspartate aminotransferase [U/l]	33.0 [24.0]
Alanine transaminase [U/l]	19.0 [34.0]
White blood cell count [/μl]	6.0 [2.7]
Hemoglobin [g/dl]	10.5 [2.6]
Platelets [/μl]	234.0 [117.0]
Lu-PSMA therapy	
Number of Lu-PSMA cycles	3 (range: 1–12)
Time between PSMA PET and Lu-PSMA start [days]	32 [22]
Average activity per cycle [GBq]	6.7 [1.2]
Cumulated activity [GBq]	21.2 [18.9]

Values are presented as median (inter quartile range) or frequency (percentage of all patients); *PSMA* prostate-specific membrane antigen, *Lu-PSMA* ^177^Lu-PSMA-617 therapy

### Correlation of non-image parameters and PSMA PET derived parameters

Lesion number (*ρ* = 0.589, *p* < 0.001), PSMA-TV (*ρ* = 0.617, *p* < 0.001), PSMA-TLU (*ρ* = 0.525, *p* < 0.001), and PSMA-TLQ (*ρ* = 0.608, *p* < 0.001) showed statistically significant correlations with prostate-specific antigen (PSA) levels. Correlations with lactate dehydrogenase were not as strong as correlations with PSA levels (Table 2).

**Table 2:** Correlation of PSMA PET parameters and blood parameters

Parameter	Prostate-specific antigen	Lactate dehydrogenase
Lesion number	0.589; *P* < 0.001	0.317; *P* = 0.001
PSMA-TV	0.617; P < 0.001	0.363; *P* < 0.001
PSMA-TLU	0.525; P < 0.001	0.230; *P* = 0.016
PSMA-TLQ	0.608; P < 0.001	0.396; P < 0.001

Spearman correlations are shown. *PSMA-TV* PSMA tumor volume, *PSMA-TLU* PSMA tumor volume multiplied by *SUV*_mean_, *PSMA-TLQ* PSMA tumor volume divided by *SUV*_mean_

### PSMA PET derived parameters and overall survival

Log transformed number of lesions was a statistically significant prognosticator of overall survival in univariate Cox regression (*p* = 0.009; HR = 1.255; 95%CI = 1.058–1.488). The same was true for PSMA-TV (*p* = 0.005; HR = 1.299; 95%CI = 1.081–1.561), PSMA-TLU (*p* = 0.046; HR = 1.171; 95%CI = 1.003–1.367), and PSMA-TLQ (*p* = 0.002; HR = 1.326; 95%CI = 1.112–1.580).

In a stepwise backward Cox regression including log transformed PSMA-TV and lesion number, only PSMA-TV remained as significant parameter (*p* = 0.005; HR = 1.299; 95%CI = 1.081–1.561). In a stepwise backward Cox regression including log transformed PSMA-TV, lesion number, prostate-specific antigen, lactate dehydrogenase, and PSMA-TLQ, only the latter two remained significant parameters in the model (LDH (*p* = 0.011; HR = 1.632; 95%CI = 1.120–2.376), PSMA-TLQ (*p* = 0.024; HR = 1.239; 95%CI = 1.029–1.492)). Multivariate Cox regression including log transformed prostate-specific antigen, lactate dehydrogenase, and PSMA-TLQ confirmed the latter two to be significant prognosticator of survival (LDH (*p* = 0.013; HR = 1.638; 95%CI = 1.110–2.418), PSMA-TLQ (*p* = 0.043; HR = 1.244; 95%CI = 1.007–1.538)). Detailed results are shown in Table 3.

**Table 3:** Regression of baseline parameters and overall survival

Parameter	Univariate Cox regression HR [95%CI]		Multivariate Cox regression HR [95%CI]	
PET parameter				
Lesion number	1.255 [1.058–1.488]	*P* = 0.009		
PSMA-TV	1.299 [1.081–1.561]	*P* = 0.005		
PSMA-TLU	1.171 [1.003–1.367]	P = 0.046		
PSMA-TLQ	1.326 [1.112–1.580]	*P* = 0.002	1.244 [1.007–1.538]	*P* = 0.043
Blood parameter				
Prostate-specific antigen	1.147 [1.021–1.289]	*P* = 0.021	0.994 [0.861–1.147]	*P* = 0.937
Lactate dehydrogenase	1.901 [1.347–2.685]	*P* < 0.001	1.638 [1.110–2.418]	*P* = 0.013

All included parameters were log (base2) transformed. *HR* hazard ratio, *CI* confidence interval, *PSMA-TV* PSMA tumor volume, *PSMA-TLU* PSMA tumor volume multiplied by *SUV*_mean_, *PSMA-TLQ* PSMA tumor volume divided by *SUV*_mean_

### Binarized PSMA PET derived parameters and overall survival

**Fig. 1:**
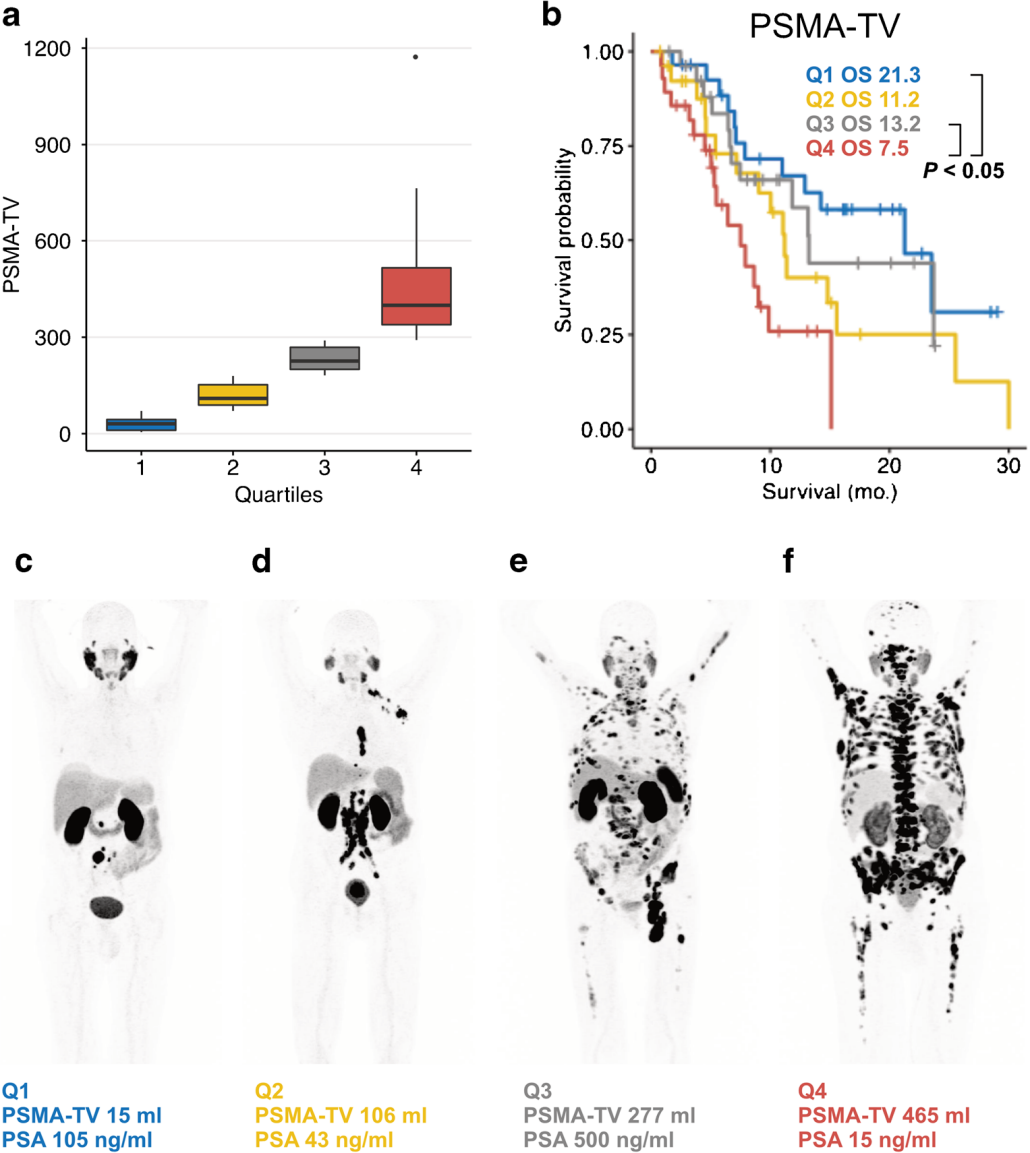
Quartiles of PSMA tumor volume and overall survival. (**a**) depicts boxplots of the PSMA tumor volume (PSMA-TV) quartile 1 to quartile 4. The overall survival is separately shown for each PSMA-TV quartile (**b**). Exemplary patients of each quartile were shown together with blood levels of prostate-specific antigen (PSA) (**c–f**)

**Fig. 2:**
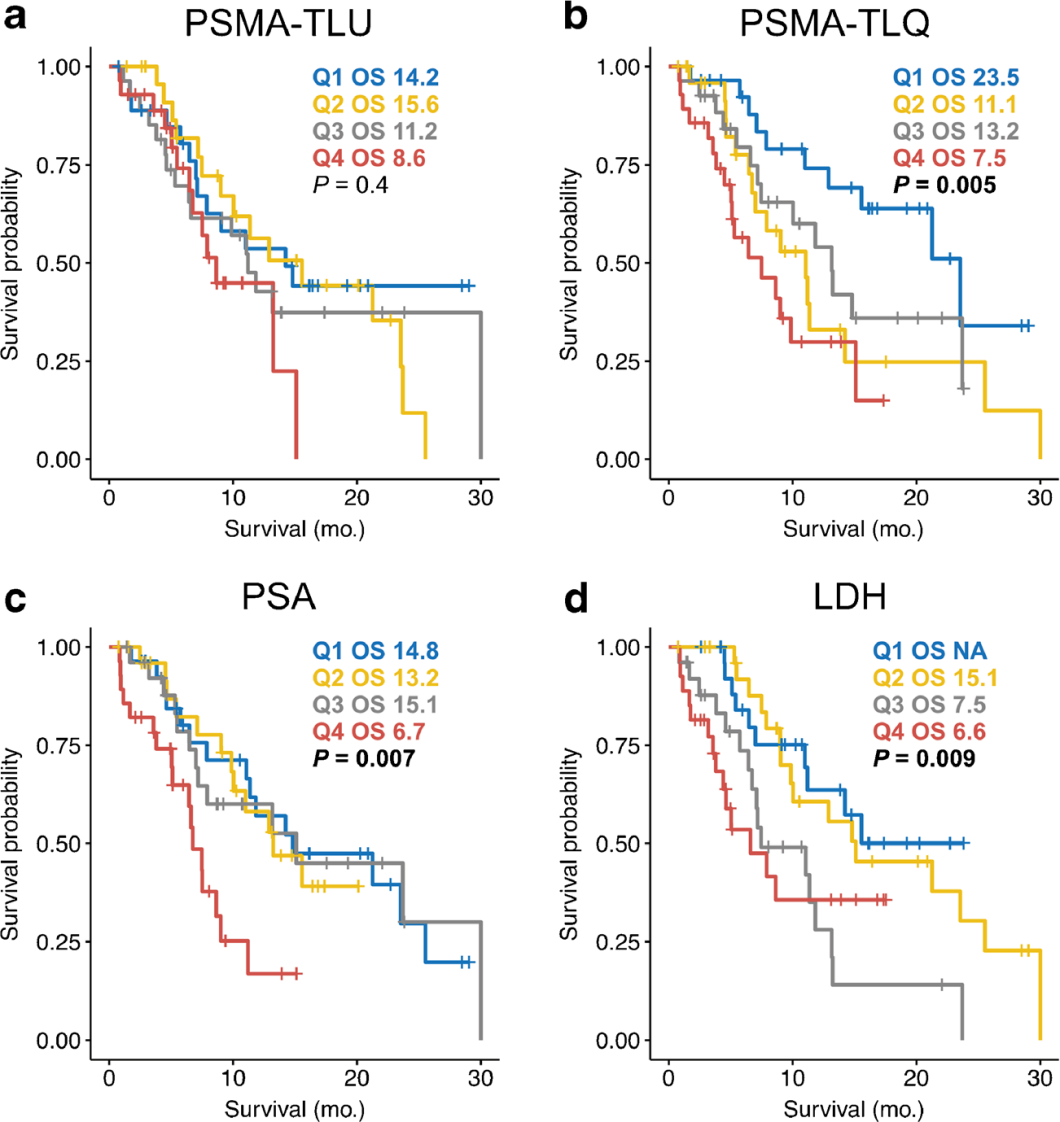
Quartiles of PET and blood biomarkers and overall survival. Patients were stratified on the basis of PSMA-TLU quartiles (**a**), PSMA-TLQ quartiles (**b**), prostate-specific antigen blood level quartiles (**c**), and lactate dehydrogenase blood level quartiles (**d**)

**Fig. 3:**
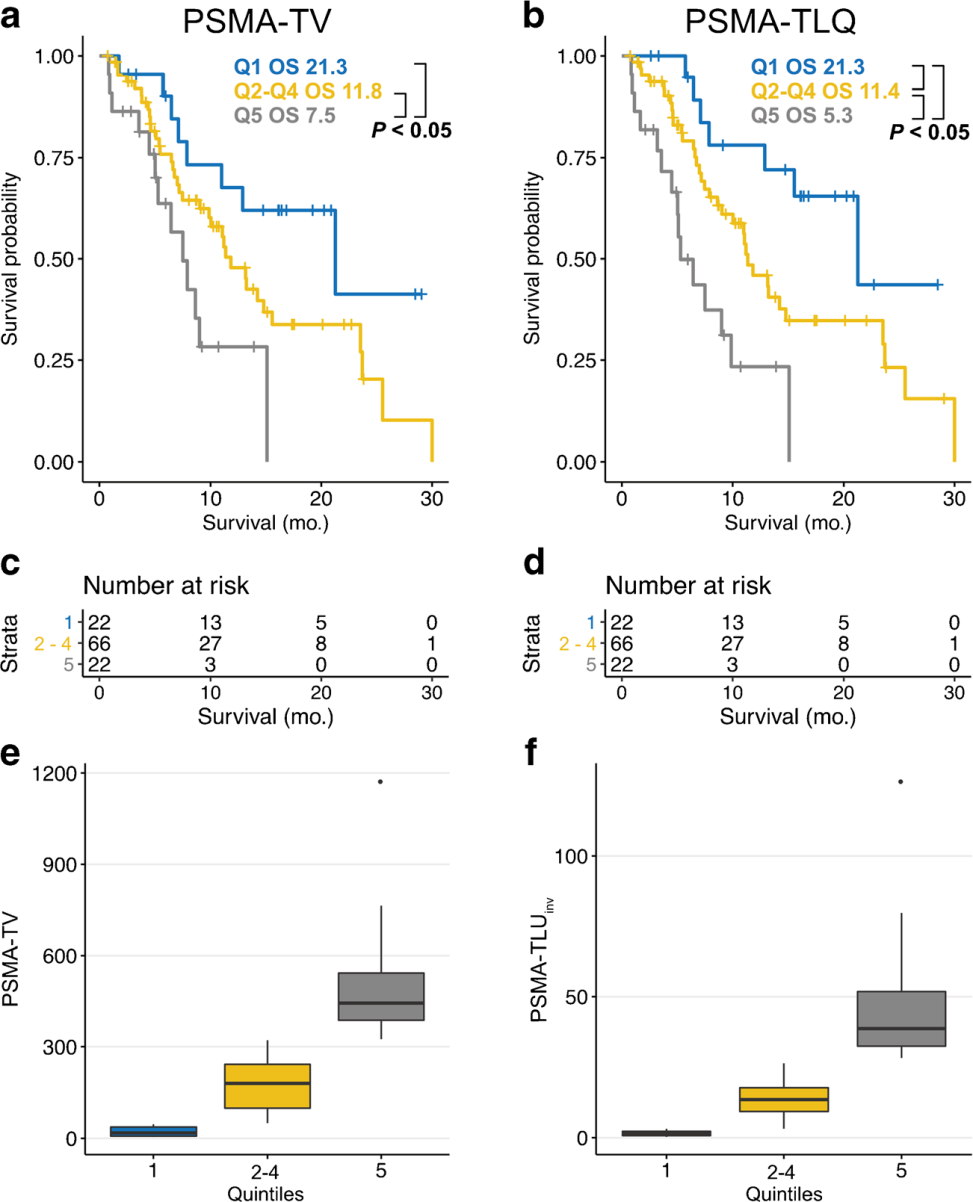
PSMA-TV and PSMA-TLQ and overall survival. Quintile 1, quintiles 2–4, and quintile 5 of PSMA-TV (**a**) or PSMA-TLQ (**b**) were employed to stratify patients and compare the median estimated overall survival between the strata. Number at risk tables were given by (**c**) and (**d**), respectively. Boxplots of quintile 1, quintiles 2–4, and quintile 4 were separately shown for PSMA-TV (**e**) and PSMA-TLQ (**f**)

Median overall survival of patients was significantly different between lesion number quartile 1 and quartile 4 (*p* = 0.02; 23.5 [95%CI: 11.0–NR] months vs. 8.6 [95%CI: 5.0–15.1] months). NR denotes not determinable values due to not reached median survival time. The same was true for quartiles 1 and 4 of PSMA-TV (*p* = 0.002; 21.3 [95%CI: 7.87–NR] months vs. 7.5 [95%CI: 5.0–9.9] months) and quartiles 1 and 4 of PSMA-TLQ (*p* < 0.001; 23.5 [95%CI: 12.9–NR] months vs. 7.5 [95%CI: 4.5–15.1] months). Median OS of quartiles 1 and 4 of PSMA-TLU were not statistically significant different from each other (*p* = 0.17; 14.2 [95%CI: 7.0–NR] months vs. 8.6 [95%CI: 6.5–NR] months). Figures 1 and 2 display the survival of stratified by the quartiles of PSMA-TV, PSMA-TLU, PSMA-TLQ, LDH, and PSA. Median overall survival of patients in PSMA-TLQ quintile 1 (21.5 [95%CI: 12.9–NR] months), quintiles 2–4 (11.4 [95%CI: 8.6–14.8] months), and quintile 5 (5.3 [95%CI: 3.6–9.9] months) were significantly different from each other (global (*p* < 0.001), quintile 1 vs. 4 (*p* < 0.001), quintiles 2–4 vs. 5 (*p* = 0.0042), quintiles 2–4 vs. 1 (0.040)). Figure 3 displays the stratification of PSMA-TV and PSMA-TLQ side by side.

**Fig. 4:**
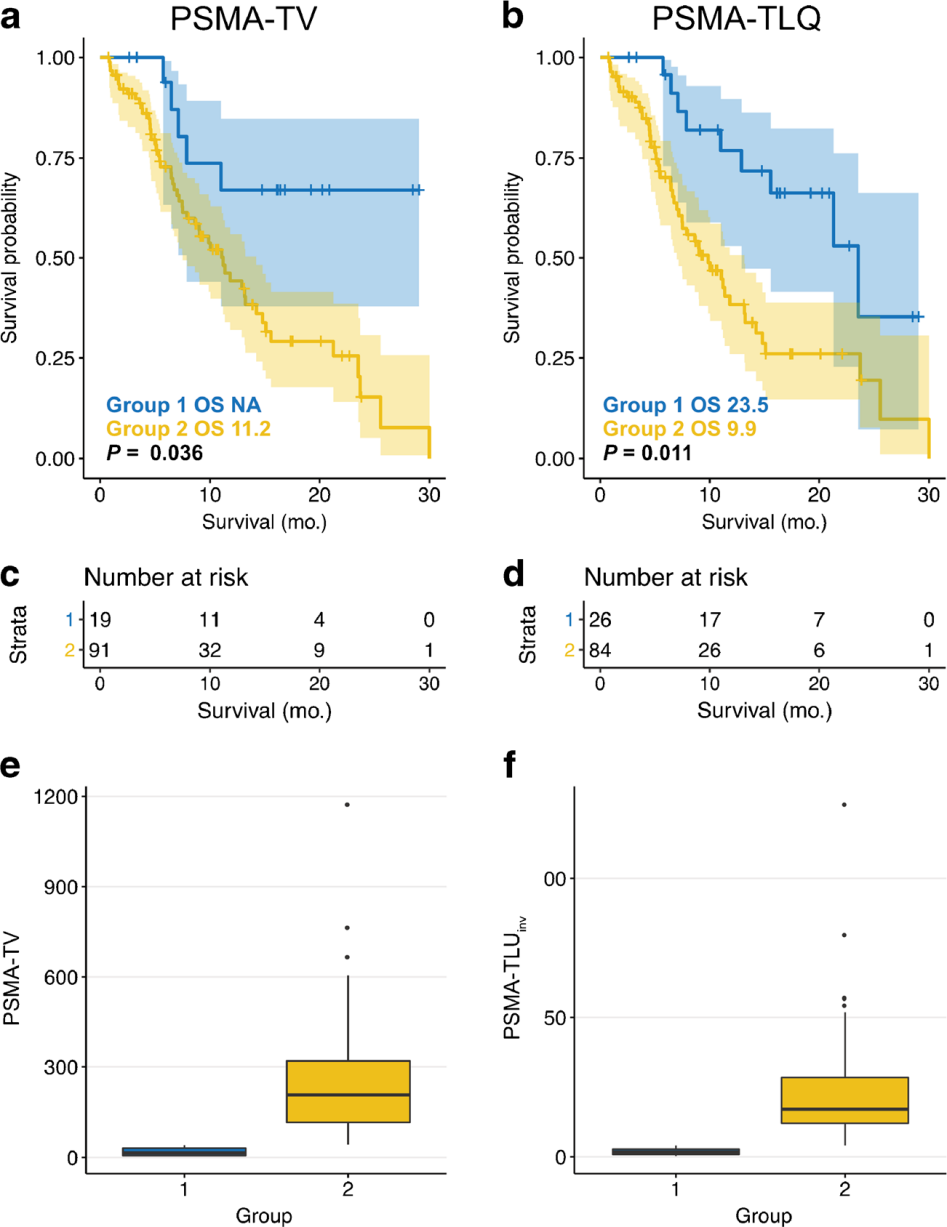
Stratification of PSMA-TV or PSMA-TLQ by ideal log rank cutoffs. A log rank cutoff finder was employed for PSMA-TV (**a**) or PSMA-TLQ (**b**) to form groups and compare the estimated median overall survival. Additionally, a number at risk tables were shown for PSMA-TV (**c**) and PSMA-TLQ (**d**). Boxplots of PSMA-TV (**e**) and PSMA-TLQ (**f**) were shown as well

Ideal log rank cutoffs were determined for PSMA-TLQ (cutoff: 4.1; *p* = 0.011; 23.5 vs. 9.9 months), prostate-specific antigen (cutoff: 514; *p* = 0.014; 14.8 vs. 7.2 months), and other parameters (see Table 4). Figure 4 displays the Kaplan Meier curves according to ideal log rank cutoffs.

**Table 4:** Binarized baseline parameters and overall survival

Parameter	Threshold	Above threshold OS [95%CI]	Below threshold OS [95%CI]	*p* Value
Lesion number	9	NR [11.0–NR]; *n* = 16	11.1 [7.5–13.2]; *n* = 94	0.004
PSMA-TV	41.1	NR [7.8–NR]; *n* = 19	11.2 [7.5–14.2]; *n* = 91	0.036
PSMA-TLU	495.2	NR [7.9–NR]; *n* = 18	11.1 [7.5–13.2]; *n* = 92	0.015
PSMA-TLQ	4.1	23.5 [12.9–NR]; *n* = 26	9.9 [7.0–13.2]; *n* = 84	0.011
Prostate-specific antigen	514	14.8 [11.4–25.5]; *n* = 77	7.2 [5.1–8.6]; *n* = 33	0.014
Lactate dehydrogenase	316	15.6 [11.0–NR]; *n* = 54	7.5 [5.7–11.8]; *n* = 55	0.020

*NR* not available due to not reached median survival, *CI* confidence interval, *PSMA-TV* PSMA tumor volume, *PSMA-TLU* PSMA tumor volume multiplied by *SUV*_mean_, *PSMA-TLQ* PSMA tumor volume divided by *SUV*_mean_

### Comparison of the Münster and Essen cohort

**Fig. 5:**
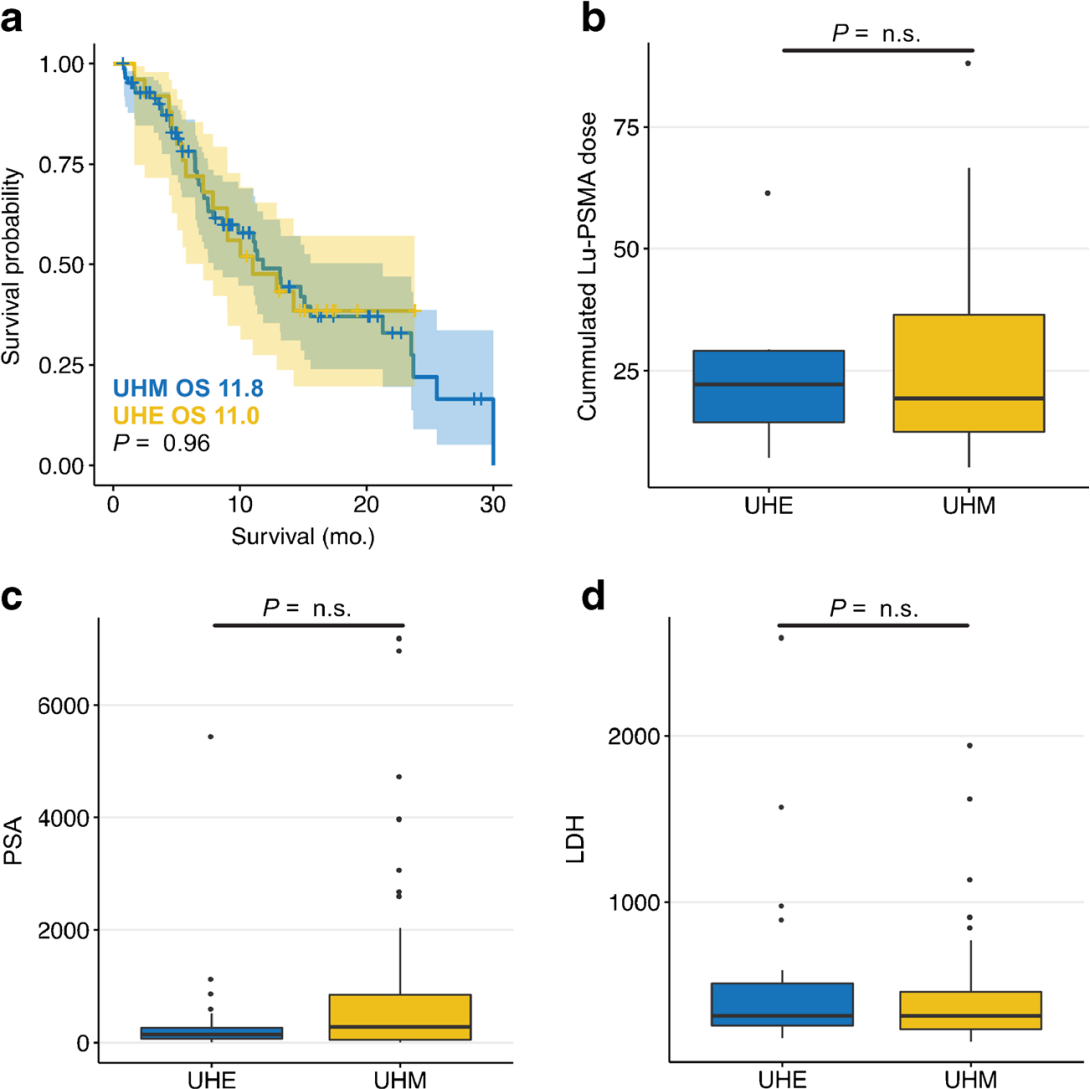
Baseline parameters and survival of Münster and Essen cohort. Patients form the university hospital Münster (UHM) or Essen (UHE) were compared with regard to overall survival (**a**), cumulated Lu-PSMA dose (**b**), prostate-specific antigen blood levels (**c**), and lactate dehydrogenase blood levels (**d**). No statistically significant differences were observed

There was no statistically significant difference regarding overall survival between the Münster and the Essen cohort (11.8 vs. 11.0 months, *p* = 0.96; HR = 0.984; 95%CI = 0.542–1.785; *p* = 0.957; see Fig. 4). Comparing the Münster and Essen cohort, the cumulated Lu-PSMA activity (19.3 vs. 22.2; *p* = 0.853), lactate dehydrogenase (316.5 vs. 317.0; *p* = 0.332), and prostate-specific antigen (284.0 vs. 145.0; *p* = 0.254) were not significantly different (see Fig. 5).

### Tumor volume and metastases location

PSMA-TV was a statistically significant negative prognosticator (HR = 1.236; 95%CI = 1.025–1.490; *p* = 0.027) in a multivariate Cox regression adjusted for presence of visceral (liver, lung, and/or pleura) (HR = 1.630; 95%CI = 0.950–2.795; *p* = 0.076), lymph node (HR = 1.060; 95%CI = 0.543–2.068; *p* = 0.865), or bone metastases (HR = 1.726; 95%CI = 0.587–5.071; *p* = 0.321). Likewise, PSMA-TLQ was a statistically significant negative prognosticator (HR = 1.278; 95%CI = 1.064–1.535; *p* = 0.009) in a multivariate Cox regression adjusted for the presence of visceral (liver, lung, and/or pleura) (HR = 1.640; 95%CI = 0.959–2.803; *p* = 0.071), lymph node (HR = 1.153; 95%CI = 0.590–2.253; *p* = 0.678), or bone metastases (HR = 1.601; 95%CI = 0.540–4.747; *p* = 0.396).

## Discussion

The present study evaluated PSMA tumor volume as biomarker to prognosticate the outcome of patients with mCRPC who were receiving Lu-PSMA therapy. To this end, the total tumor volume and related parameters were quantified in the baseline PSMA PET-CT acquisition prior to the first administration of Lu-PSMA therapy. The total tumor volume was a statistically significant negative prognosticator of survival. The integration of SUV and total tumor volume (PSMA-TLQ) leads to an improved stratification of patients according to outcome. Finally, PSMA-TLQ remained an independent prognosticator of survival in a multivariate regression including LDH and PSA blood levels.

It was shown previously that the total osseous tumor volume of patients with prostate cancer is a negative prognosticator of overall survival [11]. Moreover, Armstrong et al. could show that the risk of death increased with each quartile of osseous tumor volume [11]. The total osseous tumor volume could therefore be employed to identify homogeneous groups of patients, which might benefit from altered therapy regimes. However, as visceral metastases are also frequently present in prostate cancer patients and are associated with a poor outcome, a total tumor volume metric could be an even better prognosticator of overall survival in prostate cancer patients [1].

Other biomarkers have been employed to assess the disease extent and prognosis of prostate cancer patients. For example, the PCWG guideline recommends measuring prostate-specific antigen (PSA) blood levels to assess biochemical response to therapy [22]. However, the association of PSA levels and the total tumor volume might be hampered by androgen deprivation therapy and more importantly prostate cancer heterogeneity together with treatment-induced dedifferentiation [18, 23]. This is in line with the findings of the present study. PSA was not a statistically significant prognosticator of overall survival, which is indicating its limited value to monitor therapy response. Thus, therapeutic decisions in mCRPC should not be based on PSA but on imaging data and clinical symptoms.

Due to the detection of both bone and soft tissue metastases, PSMA PET derived total tumor volume might be superior to bone scan derived total tumor volume [24]. Therefore, several approaches have been proposed to quantify the total tumor volume in PSMA PET: Gafita et al. had proposed qPSMA, a software which segments all malignancy suspicious lesions in PSMA PET acquisitions based on a liver specific threshold [19]. Ferdinandus et al. had proposed a method in which all lesions were segmented by employing an SUV threshold of 3 [12]. Seifert et al. had proposed a two-step approach in which a liver specific threshold was employed for lesion selection and a regional threshold (50% of the local *SUV*_max_) for lesion segmentation [13]. In the present study, the quantification approach of Seifert et al. was employed [13].

There are diverting reports on the prognostic value of PSMA PET derived total tumor volume in patients with end-stage prostate cancer who receive Lu-PSMA therapy: Ferdinandus et al. could show that the FDG PET derived total tumor volume and the bone scan derived tumor volume were negative prognosticators of survival; yet the PSMA PET derived tumor volume was not a statistically significant prognosticator in their study [12]. This finding seems counter intuitive, as greater tumor volume is generally linked with worse outcome. Seifert et al. could show that the tumor volume is a negative prognosticator of overall survival in a preliminary analysis [13]. Here, we corroborated these findings by broadening the patient collective and including patients from a second department. PSMA-TV was a statistically significant negative prognosticator of overall survival. In line with the findings from the bone scan tumor volume analyses, the PSMA-TV parameter was superior to the lesion number as a prognosticator of overall survival in patients with end-stage prostate cancer.

It was shown previously that *SUV*_mean_ is a positive prognosticator of survival for patients with end-stage prostate cancer receiving Lu-PSMA therapy [12, 15]. In line with the concept of theranostics, high *SUV*_mean_ is associated with higher tumor doses of Lu-PSMA therapy and should therefore be linked to a favorable outcome [25]. Therefore, the integration of tumor volume and SUV to form a combined biomarker seems reasonable, which was done in analogy to FDG total lesion glycolysis by Seifert et al. and Gafita et al. [13, 19]. However, as the tumor volume is a negative prognosticator and SUV uptake a positive prognosticator, both metrics may antagonize in the PSMA-TLU biomarker. Therefore, a novel biomarker (PSMA-TLQ) was proposed by this study, which is the quotient of total tumor volume and *SUV*_mean_. As expected, PSMA-TLQ was a better prognosticator of survival compared with lesion number and even to PSMA-TV. Interestingly, PSMA-TLQ remained a significant prognosticator of overall survival, even if the regression was adjusted for the presence of visceral, bone, and lymph node metastases. Therefore, it might be warranted to evaluate PSMA-TLQ in future studies and determine if the weighting between tumor volume and SUV can be optimized.

The estimated overall survival of patients significantly decreased with increasing PSMA-TLQ. Interestingly, the overall survival between quintile 1, quintiles 2–4, and quintile 5 were statistically significant different. Quintiles 2 to 4 were pooled, as these were not adequately separating groups with distinct median overall survival, which was likely due to the limited sample size. Stratification of patients according to quintiles of a radiographic biomarker is in line with the findings of Armstong et al. regarding the bone scan tumor volume [11]. Therefore, the PSMA-TLQ biomarker might be used to identify homogenous patient cohorts for treatment intensification.

PSMA-TLQ was a statistically significant prognosticator of overall survival; the metric integrates the tumor volume and *SUV*_mean_. As mentioned above, higher *SUV*_mean_ is generally associated with higher dose delivery through PSMA targeted therapy [25]. If more aggressive prostate cancer cell phenotypes evolve, the PSMA expression might decrease [26]. Because of that, FDG PET is sometimes performed in addition to PSMA PET, which helps to detect dedifferentiated metastases [12]. To date, it remains unclear if the favorable outcome of end-stage patients with a strong PSMA expression is due to higher dose delivery, lesser dedifferentiation, or a combination of both. Interestingly, high PSMA expression can likewise be a marker of aggressive tumor phenotypes. Especially in the primary staging of prostate cancer, strong PSMA expression is associated with higher Gleason scores [27]. However, the implications of in vivo PSMA tracer uptake are poorly elucidated in a castration resistant and metastatic situation.

The present analysis had some limitations. The analysis was performed retrospectively and is therefore prone to selection biases. Alkaline phosphatase levels were not present for all patients. Moreover, the tumor volume was determined as whole-body metric and not in an organ-wise manner. As visceral metastases are associated with shorter overall survival time, future studies should quantify an organ system wise tumor volume. Additionally, the sample size was limited, which might hamper generalizability. However, to minimize those effects, a bicentric approach was chosen. No FDG PET was included in the analysis as it was not implemented in the clinical routine for the included patients. The volume of dedifferentiated tumor parts that could not adequately be captured by PSMA PET might have been correctly quantified by FDG PET. Therefore, future studies integrating the findings of FDG PET and PSMA PET are warranted. These studies should also evaluate PSMA-TV and PSMA-TLQ changes over time to establish both as response parameters.

## Conclusion

PSMA tumor volume was a negative prognosticator of survival in patients treated with Lu-PSMA therapy. PSMA-TLQ, a metric integrating PSMA tumor volume and PET uptake, was a statistically independent prognosticator of survival in a multivariate analysis and might be a new PET based metric to prognosticate response to radioligand therapies. Future studies are warranted to corroborate these findings in end-stage prostate cancer patients treated with other systemic therapies.

## References

[1] Sartor O, de Bono JS (2018). Metastatic prostate cancer. N Engl J Med.

[2] Rahbar K, Ahmadzadehfar H, Kratochwil C, Haberkorn U, Schäfers M, Essler M (2017). German multicenter study investigating ^177^ Lu-PSMA-617 radioligand therapy in advanced prostate cancer patients. J Nucl Med.

[3] Hofman MS, Violet J, Hicks RJ, Ferdinandus J, Ping Thang S, Akhurst T, et al. [177 Lu]-PSMA-617 radionuclide treatment in patients with metastatic castration-resistant prostate cancer (LuPSMA trial): a single-centre, single-arm, phase 2 study. Lancet Oncol. 2018;19(6):825–33. 10.1016/S1470-2045(18)30198-0 29752180

[4] Seifert R, Kessel K, Schlack K, Weckesser M, Bögemann M, Rahbar K. Radioligand therapy using [177Lu]Lu-PSMA-617 in mCRPC: a pre-VISION single-center analysis. Eur J Nucl Med Mol Imaging. 2020;47(9):2106–12. 10.1007/s00259-020-04703-3 PMC7338828 32062682

[5] Hofman MS, Emmett L, Violet J, Zhang AY, Lawrence NJ, Stockler M (2019). TheraP: a randomized phase 2 trial of 177Lu-PSMA-617 theranostic treatment vs cabazitaxel in progressive metastatic castration-resistant prostate cancer (Clinical Trial Protocol ANZUP 1603). BJU Int.

[6] Rahbar K, Bodei L, Morris MJ. Is the “VISION” of radioligand therapy for prostate cancer becoming reality? an overview of the phase III trial and the importance for the future of theranostics. J Nucl Med. 2019. 10.2967/jnumed.119.234054. 10.2967/jnumed.119.234054 PMC12076095 31451487

[7] Ahmadzadehfar H, Rahbar K, Kürpig S, Bögemann M, Claesener M, Eppard E (2015). Early side effects and first results of radioligand therapy with (177)Lu-DKFZ-617 PSMA of castrate-resistant metastatic prostate cancer: a two-centre study. EJNMMI Res.

[8] Hofman M, Emmett L, Violet JA, Lawrence NJ, Williams S, Stockler MR (2019). TheraP: a randomized phase II trial of [177Lu]-PSMA-617 theranostic versus cabazitaxel in progressive metastatic castration-resistant prostate cancer. J Clin Oncol.

[9] Sweeney CJ, Chen Y-H, Carducci M, Liu G, Jarrard DF, Eisenberger M (2015). Chemohormonal therapy in metastatic hormone-sensitive prostate cancer. N Engl J Med.

[10] Kyriakopoulos CE, Chen Y-H, Carducci MA, Liu G, Jarrard DF, Hahn NM (2018). Chemohormonal therapy in metastatic hormone-sensitive prostate cancer: long-term survival analysis of the randomized phase III E3805 CHAARTED trial. J Clin Oncol.

[11] Armstrong AJ, Anand A, Edenbrandt L, Bondesson E, Bjartell A, Widmark A (2018). Phase 3 assessment of the automated bone scan index as a prognostic imaging biomarker of overall survival in men with metastatic castration-resistant prostate cancer a secondary analysis of a randomized clinical trial. JAMA Oncol.

[12] Ferdinandus J, Violet J, Sandhu S, Hicks RJ, Ravi Kumar AS, Iravani A, et al. Prognostic biomarkers in men with metastatic castration-resistant prostate cancer receiving [177Lu]-PSMA-617. Eur J Nucl Med Mol Imaging. 2020;47:2322–7. 10.1007/s00259-020-04723-z 32140802

[13] Seifert R, Herrmann K, Kleesiek J, Schafers MA, Shah V, Xu Z, et al. Semi-automatically quantified tumor volume using Ga-68-PSMA-11-PET as biomarker for survival in patients with advanced prostate cancer. J Nucl Med. 2020. 10.2967/jnumed.120.242057 [Internet]. Available from: http://jnm.snmjournals.org/lookup/doi/10.2967/jnumed.120.242057. 10.2967/jnumed.120.242057 32332147

[14] Kratochwil C, Fendler WP, Eiber M, Baum R, Bozkurt MF, Czernin J (2019). EANM procedure guidelines for radionuclide therapy with 177Lu-labelled PSMA-ligands (177Lu-PSMA-RLT). Eur J Nucl Med Mol Imaging.

[15] Seifert R, Seitzer K, Herrmann K, Kessel K, Schäfers M, Kleesiek J, et al. Analysis of PSMA expression and outcome in patients with advanced prostate cancer receiving 177Lu-PSMA-617 radioligand therapy. Theranostics. 2020;10(17):7812–20. 10.7150/thno.47251 PMC7359095 32685021

[16] Gafita A, Fendler WP, Hui W, Sandhu S, Weber M, Esfandiari R, et al. Efficacy and safety of 177Lu-labeled prostate-specific membrane antigen radionuclide treatment in patients with diffuse bone marrow involvement: a multicenter retrospective study. Eur Urol. 2020;78(2):148–54. 10.1016/j.eururo.2020.05.004 32532512

[17] Rahbar K, Bode A, Weckesser M, Avramovic N, Claesener M, Stegger L (2016). Radioligand therapy with 177Lu-PSMA-617 as a novel therapeutic option in patients with metastatic castration resistant prostate cancer. Clin Nucl Med.

[18] Denmeade SR, Sokoll LJ, Dalrymple S, Rosen DM, Gady AM, Bruzek D (2003). Dissociation between androgen responsiveness for malignant growth vs. expression of prostate specific differentiation markers PSA, hK2, and PSMA in human prostate cancer models. Prostate..

[19] Gafita A, Bieth M, Krönke M, Tetteh G, Navarro F, Wang H (2019). qPSMA: semiautomatic software for whole-body tumor burden assessment in prostate cancer using 68 Ga-PSMA11 PET/CT. J Nucl Med.

[20] R Core Team (2018). R: a language and environment for statistical computing [Internet].

[21] Hothorn T, Lausen B (2003). On the exact distribution of maximally selected rank statistics. Comput Stat Data Anal.

[22] Scher HI, Morris MJ, Stadler WM, Higano C, Basch E, Fizazi K (2016). Trial design and objectives for castration-resistant prostate cancer: updated recommendations from the prostate cancer clinical trials working group 3. J Clin Oncol.

[23] Aggarwal R, Huang J, Alumkal JJ, Zhang L, Feng FY, Thomas GV (2018). Clinical and genomic characterization of treatment-emergent small-cell neuroendocrine prostate cancer: a multi-institutional prospective study. J Clin Oncol.

[24] Halabi S, Kelly WK, Ma H, Zhou H, Solomon NC, Fizazi K (2016). Meta-analysis evaluating the impact of site of metastasis on overall survival in men with castration-resistant prostate cancer. J Clin Oncol.

[25] Violet J, Jackson P, Ferdinandus J, Sandhu S, Akhurst T, Iravani A (2019). Dosimetry of 177Lu-PSMA-617 in metastatic castration-resistant prostate cancer: correlations between pretherapeutic imaging and whole-body tumor dosimetry with treatment outcomes. J Nucl Med.

[26] Bakht MK, Derecichei I, Li Y, Ferraiuolo RM, Dunning M, Oh SW (2019). Neuroendocrine differentiation of prostate cancer leads to PSMA suppression. Endocr Relat Cancer.

[27] Uprimny C, Kroiss AS, Decristoforo C, Fritz J, von Guggenberg E, Kendler D (2017). 68Ga-PSMA-11 PET/CT in primary staging of prostate cancer: PSA and Gleason score predict the intensity of tracer accumulation in the primary tumour. Eur J Nucl Med Mol Imaging.

